# Nanotechnology-Based Drug Delivery for Parkinson’s Disease: A Review On Strategies, Efficacy and Translational Hurdles

**DOI:** 10.34172/apb.46162

**Published:** 2026-05-09

**Authors:** Meghas Hari, Abhirami Subramony, Deepak Dwivedi, Reshmi S Krishnan, Aswin Damodaran, Sreeja C Nair

**Affiliations:** Amrita School of Pharmacy, Amrita Vishwa Vidyapeetham, AIMS Health Sciences Campus, Kochi -682041, India

**Keywords:** Parkinson’s disease, Nanotechnology, Drug delivery, Nanoparticles, Targeted therapy

## Abstract

Treatment for Parkinson’s disease (PD) faces two significant challenges: first, the blood-brain barrier (BBB) and second, a therapeutic strategy that alleviates symptoms instead of stopping the disease progression. The BBB prevents over 98% of small-molecule drugs, leaving neuroprotective options mostly inaccessible. Nanotechnology is being developed to overcome existing limitations, paving the way for more precise and effective delivery to the brain. This review goes beyond listing types of nanocarriers to critically synthesise our understanding of nanoparticle (NP)-based strategies for PD, evaluating their therapeutic potential in relation to the challenges of real-world application. Evidence from various systems is evaluated, from lipid-based carriers and polymeric NPs to advanced constructs such as dendrimers, to see how these might enhance brain bioavailability, support sustained release, and directly target disease processes, including alpha-synuclein aggregation and neuroinflammation. The paper thoroughly examines the primary barriers to clinical application, including biocompatibility concerns, scalability of manufacturing, and an evolving regulatory landscape. Looking ahead, PD nanomedicine will focus on designing theranostic platforms with multiple functions. That future will depend upon close collaborations across disciplines to turn compelling preclinical results into practical, disease-modifying therapies.

## Introduction

 Parkinson’s disease (PD) is a widespread neurodegenerative disorder marked by the gradual loss of dopaminergic neurones in the substantia nigra pars compacta of the brain, as well as the development of intracellular protein aggregates known as Lewy bodies, predominantly composed of aggregated α-synuclein protein.^1^ The consequent lack of dopamine within the striatum gives rise to the prominent motor symptoms of PD, such as bradykinesia, rest tremor, rigidity, and postural instability, which significantly impair patients’ quality of life. Since its introduction over half a century ago, Levodopa, a precursor to dopamine, has remained the first-line drug for treating PD. Although effective for alleviating symptoms, its repeated administration over time results in motor complications such as dyskinesia and a reduced therapeutic window.^2,3^ Notably, Levodopa and all available therapies have failed to slow the neurodegenerative component of PD, thus identifying the urgent demand for therapies aimed at both alleviating symptoms and addressing the disease’s root pathophysiology, including α-synuclein aggregation or mitochondrial defects.^4^

 The obstacle of crossing the blood-brain barrier (BBB) is a significant challenge for disease-modifying therapeutic approaches. The BBB is a very selectively permeable barrier that restricts the passage of more than 98% of small molecule therapeutics and all large biologics to the brain.^5^ However, the application of nanotechnology holds great potential for bypassing delivery difficulties.^6^ Engineered nanoparticles (NPs) can be designed to enhance the solubility of the therapeutic, prolong the circulating half-life, and target the drug to the brain, as shown in Figure 1.^7^ Several types of nanocarriers, such as liposomes and polymeric NPs, have shown enhanced brain delivery and efficacy in preclinical models of PD. However, it is a significant challenge to translate these preclinical observations to human therapies that can be approved for clinical use.^8^

**Figure 1:**
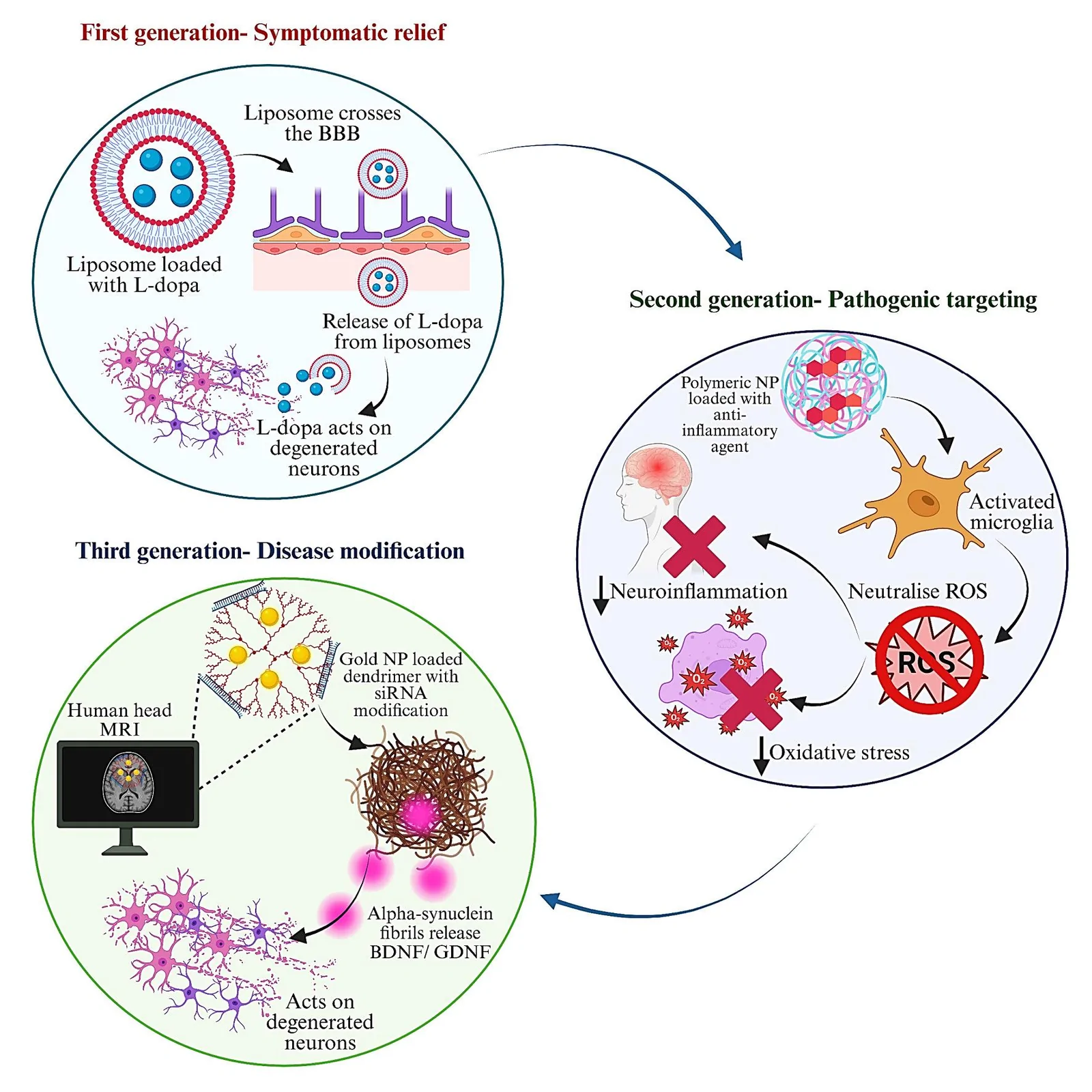
Evolution of nanotechnology in the treatment of PD

 This review provides a critical perspective of the therapeutic approaches based on NPs for PD treatment.^9^ It summarises and critically assesses the preclinical information available for different nanocarriers regarding their advantages and disadvantages to highlight the important aspects that need to be translated to realise the potential of nanomedicine for PD treatment.^10^

## Pathogenesis of Parkinson’s Disease

 PD pathogenesis is now understood to be a complex process of interplay between genetic factors and environmental components, as well as a variety of intercellular interactions that finally destroy the dopaminergic neurons of the substantia nigra compacta region of the brain.^11^ This results in a significant deficit of dopamine levels, which is responsible for the key motor symptoms of PD. Hallmark changes of Central PD are the deposition of α-synuclein aggregates into what is termed Lewy bodies in the brain. This is coupled with mitochondrial dysfunction.

###  Genetic and Environmental Predisposition

 The disease is predominantly sporadic, although it has a family history in 10 to 15% of affected persons. Investigating genes linked to inherited variants, SNCA (α-synuclein gene), LRRK2, PARKIN, PINK1, and DJ1, has played a crucial role in understanding the molecular pathology of PD.^12,13^ These genes are involved in pathways that, if disrupted, are characteristic of non-inherited PD, and include protein aggregation, mitochondrial, and oxidative stress pathways. Environmental exposures, pesticide exposure (rotenone and paraquat), and heavy metals have played a major role and have shown a strong epidemiologic association with the risk of developing PD.^14^ They are assumed to induce both mitochondrial damage and oxidative stress. It is assumed to occur in response to an individual’s predisposed exposure to his/her surroundings, triggering the disease.^15^

###  Alpha-Synuclein Aggregation and Propagation 

 α-synuclein is a presynaptic protein and plays a key role in the pathophysiology of PD. In physiological conditions, α-synuclein is involved in neuronal transmission.^16^ In PD, however, it undergoes misfolding and accumulation, and the process begins with a small soluble oligomer, which is extremely toxic, and eventually leads to a larger, insoluble fibril, giving rise to Lewy body inclusions.^17,18^ The primary features of Parkinson’s disease progression include the prion-like transmission of α-synuclein, wherein the abnormal protein is secreted by pathological neurons and taken up by healthy neurons, thus transmitting the disease throughout the body.^19^ Mutations in SNCA accelerate the protein-aggregation process and thus play a crucial role in disease progression.^20^

###  Mitochondrial Dysfunction and Oxidative Stress

 There is substantial evidence that the dopaminergic neurons in PD are particularly sensitive to mitochondrial dysfunction. A key finding that has been consistently reported is the reduction in mitochondrial complex I activity in the substantia nigra in PD. This is because there is a reduction in ATP production; in due course, the levels of ROS are increased, thus inducing persistent oxidative stress.^21,22^ Oxidative stress can damage proteins, lipids, as well as DNA, thus inducing further dysfunction in the cells.^23^ Furthermore, the machinery that is responsible for the maintenance of healthy mitochondria will be affected, thus inducing dysfunctional mitochondria.^24^ Mitochondrial damage is highlighted by the association between genetic PD and mitophagy-related genes, which include PINK1/PARKIN.^25,26^

###  Neuroinflammation 

 Progression in PD is well known to involve a perpetual activation of the innate immune response in the brain.^27^ The activation of microglial cells and astrocytes is a result of α-synuclein aggregation and neuronal damage.^28^ It gives rise to a pro-inflammatory environment because they secrete pro-inflammatory factors like TNF-α, IL-1β, and IL-6, which are neurotoxic. This neuroinflammatory response is a contributory factor to neuronal damage but is also a perpetuator of a vicious cycle because it gives rise to more α-synuclein aggregation, leading to mitochondrial damage and neuronal loss.^29^

###  Converging Pathways and Therapeutic Implications 

 A vicious cycle of pathology in PD is initiated by protein aggregation, mitochondrial dysfunction, and neuroinflammation.^30^ For example, mitochondrial dysfunction may lead to α-synuclein aggregation, which in turn results in further neuroinflammation and mitochondrial impairment. Such a complex pathology renders the treatment of PD rather difficult. Nowadays, there is an increased requirement for pharmaceuticals that possess the capacity to target multiple pathways concurrently.^31^ However, the BBB continues to remain a daunting challenge, which restricts many drugs from entering the brain. At this stage, NP delivery systems prove essential, facilitating the transport of therapeutics across the barrier to the target site of action.

## Current Treatment Landscape and the Imperative for Nanotechnology

 Management of PD remains one of the greatest clinical challenges. The standard of care combines drug therapy, lifestyle and rehabilitation approaches, and surgery in selected cases. These approaches, however, predominantly address symptoms rather than disease modification, and each has significant adverse effects impacting daily life for patients and does not slow disease progression.^32^

###  Limitations of Symptomatic Pharmacological and Surgical Therapies

 The pharmacokinetic properties of key PD drugs help explain many of their failings, as illustrated in Table 1.

**Table 1:** Pharmacokinetic parameters of currently used medications in PD therapy.

**Sl No**.	**Drug (Route)**	**Key PK parameters**	**Major limitations**	**Ref**
1.	Levodopa/Carbidopa (oral)	T_max_: 0.5-2.0 h; C_max_: 1.5-5.0 µg/mL (after 100-250 mg levodopa); t_½_: ~1.5-2.0 h (with carbidopa); Bioavailability: ~15-33%	Short half-life leading to motor fluctuations; erratic gastric absorption	^33^
2.	Levodopa/Carbidopa-CR (oral)	T_max_: 2.0-4.0 h; C_max_: lower than IR on a dose-normalised basis (~30-50% lower); t_½_: apparent prolonged but elimination similar (~1.5-2.5 h); Bioavailability extent ≈ 70-75% of IR.	Delayed onset; variable absorption; less predictable plasma peaks	^34^
3.	Pramipexole (oral)	T_max_: ~2-3 h; t_½_: ~8 h (young) - ~12 h (elderly); Bioavailability: > 90%	Less effective than levodopa for motor symptoms; impulse control disorders, somnolence	^35^
4.	Ropinirole (oral)	T_max_: 1-2 h (IR); t_½_: ~6 h; Bioavailability: ~50% (formulation-dependent)	Similar dopamine agonist AEs; requires multiple daily dosing (IR)	^36^
5.	Rotigotine (transdermal patch)	T_max_: ~15-18 h to steady-state exposure; t_½ _(terminal): 5-7 h (after patch removal); Bioavailability: ~37% (systemic exposure)	Skin reactions at patch site; limited dose flexibility	^37^
6.	Levodopa (inhaled)	T_max_: ~10-30 min (peak ~30 min for 84 mg); t_½_: ~2.3 h (apparent terminal following inhaled dose with carbidopa pretreatment)	Approved for intermittent 'off' rescue only; not for routine maintenance; local pulmonary AEs reported	^38^
7.	Levodopa/carbidopa/entacapone (oral)	Levodopa T_max_: ~1-2 h; entacapone increases levodopa AUC ≈ ~30-40% and prolongs apparent levodopa exposure (apparent t_½_ ≈ ~2.0-2.5 h vs ~1.7 h without COMT inhibitor). Cmax is dose/formulation dependent.	Combined AE profile (levodopa-related dyskinesia, nausea) + entacapone effects (GI upset, urine discoloration); dose optimization required.	^39^
8.	Levodopa/carbidopa (intestinal gel/jejunal infusion)	Continuous jejunal infusion (Percutaneous Endoscopic Gastrostomy-Jejunal tube): steady-state plasma levels reached within ~2-3 h; no discrete C_max_ (intended to minimize peak-to-trough); intrinsic levodopa elimination t_½_ ~1.5-2.0 h unchanged; markedly reduced plasma fluctuation vs oral.	Invasive PEG-J placement; device/procedure AEs (tube dislocation, infection); specialized support and cost.	^40^
9.	Entacapone (oral adjunct)	T_max_: ~1 h; C_max_ and exposure reflect rapid absorption after 200 mg; elimination t_½_: ~0.4-0.7 h (rapid), with clinical effect via peripheral COMT inhibition increasing levodopa AUC when co-administered.	Short half-life and so must be dosed with each levodopa dose; no intrinsic antiparkinsonian benefits alone; GI AEs, urine discoloration.	^41^
10.	Tolcapone (oral)	T_max_: ~1.5-2.0 h; C_max_ and exposure dose-dependent (e.g., 100-200 mg); elimination t_½_: ~2-3 h (longer than entacapone); higher systemic exposure and central COMT inhibition.	Hepatotoxicity risk - requires liver monitoring; restricted/limited use despite longer action.	^42^
11.	Apomorphine (subcutaneous injection)	T_max_ (SC): ~10-20 min; C_max_ example: few ng/mL after typical 2-6 mg SC doses (study dependent); t_½_: ~30-60 min; bioavailability near 100% (SC).	Short duration of action; requires antiemetic (trimethobenzamide) prophylaxis; injection-site reactions; hypotension, nausea; rescue therapy only.	^43^
12	Rasagiline (oral)	T_max_: ~0.5-1.0 h; C_max_ (1 mg): ~2-8 ng/mL (reported ranges across studies); plasma t_½_: ~1.5-3.5 h (but effect outlasts plasma levels due to irreversible MAO-B inhibition); oral bioavailability ~35%.	Modest symptomatic benefit vs levodopa; drug-drug interactions typical for MAO-B inhibitors; no proven neuroprotection in humans.	^44^
13	Safinamide (oral)	T_max_: ~2-3 h; C_max_ (100 mg): ~15-25 ng/mL (study reports vary); t_½_: ~20-30 h (supports once-daily dosing); high oral bioavailability reported.	Adjunctive use only; MAO-B interaction potential at high doses; no proven disease modification.	^45^
14.	Amantadine hydrochloride (oral)	T_max_: ~2-4 h; C_max_ (100 mg): ~250-500 ng/mL (varies with formulation and age); t½: ~10-15 h (can exceed 24 h in elderly or renal impairment); oral bioavailability ~80-100%.	CNS adverse effects (confusion, hallucinations), dose adjustment needed in renal impairment, limited core motor efficacy.	^46^

 Levodopa, the most potent agent for motor control, has a short plasma half-life (~1.5–2 hours), yielding fluctuating brain levels, unpredictable “wearing-off” periods, and, with long-term use, incapacitating levodopa-induced dyskinesias.^47^ Longer-acting dopamine agonists like pramipexole and ropinirole are less potent overall and carry a significant risk of adverse non-motor effects, including impulse control disorders, hallucinations, and excessive daytime somnolence.^48^ Adjuncts such as MAO-B inhibitors and COMT inhibitors extend levodopa’s action only partially and introduce potential drug–drug and drug–diet interactions.

 In the advanced stages of PD, deep-brain stimulation can provide significant motor alleviation, but it is invasive, carrying risks of infection, bleeding, and hardware complications. It is not appropriate for all patients; it necessitates careful patient selection, is costly, and does not effectively address important non-motor symptoms or disease progression. Non-pharmacological interventions, like physical and speech therapy, are indeed essential components of comprehensive care but also present their own challenges in terms of access, affordability, and adherence, without targeting the neurodegenerative process directly.

###  The Failure of Disease-Modifying Therapies and the BBB Challenge

 The pressing need in PD is the need for a treatment that can slow down or halt the loss of dopaminergic neurons. Despite decades of research, no neuroprotective or disease-modifying therapy has made it to routine clinical use.^49^

 Novel ideas, such as gene therapy, face substantial challenges around delivery, precision targeting, and possible immune responses. The root of these problems is the BBB.^50^ Very few agents can cross this selective barrier; in fact, it blocks more than 98% of small-molecule drugs and nearly all large biologics. Agents aimed at core drivers, such as α-synuclein aggregation, mitochondrial problems, or neuroinflammation, often fail because they never reach the brain in therapeutic quantities. To achieve sufficient brain concentrations, generally, high systemic doses are required, with the consequence of unwanted peripheral side effects and complexities of the treatment regimen.^51^

###  The Rationale for Nanoparticle-Based Drug Delivery

 Given these major obstacles, there’s an urgent push toward NP-based delivery systems. Tapping into nanotechnology allows us to design advanced platforms that address the BBB and the failures in treatment modalities through the provision of:

- **Improved BBB crossing**: NPs can be engineered on the surface with ligands, peptides, and antibodies, which promote active transport via transcystosis.^52^
- **Improved pharmacokinetics**: The encapsulation protects the therapeutic cargo from degradation, prolongs circulation time, and enables controlled release, diminishing the fluctuations in dosing responsible for motor problems associated with levodopa.
- **Targeted action**: It is possible to modify NPs to target specific cell types (neurons, activated microglia) or even subcellular organelles, such as mitochondria, to concentrate the effect where it is needed, thus limiting systemic side effects.^53^
- **Combination and Advanced Therapies**: The flexible cargo space of NPs allows co-delivery of multiple agents, for example, a gene-silencing RNA with an anti-inflammatory drug and the transport of complex biologics for a coordinated attack against the multifactorial nature of PD.^54^

The following sections will critically discuss the various NP classes developed to achieve these goals, review the preclinical evidence, and provide an overview of how to translate this into the clinic.

## Critical Analysis of Nanoparticle Platforms for PD Therapy

 Having highlighted the shortcomings of existing therapies and the urgent need for target-specific delivery, the next part will review the leading NP delivery systems currently being considered for the treatment of PD (Figure 2). It will discuss the preclinical data for the ability of the delivery systems to bypass the BBB, efficacy in models of PD, and the unique benefits and challenges for each delivery system.^55^

**Figure 2:**
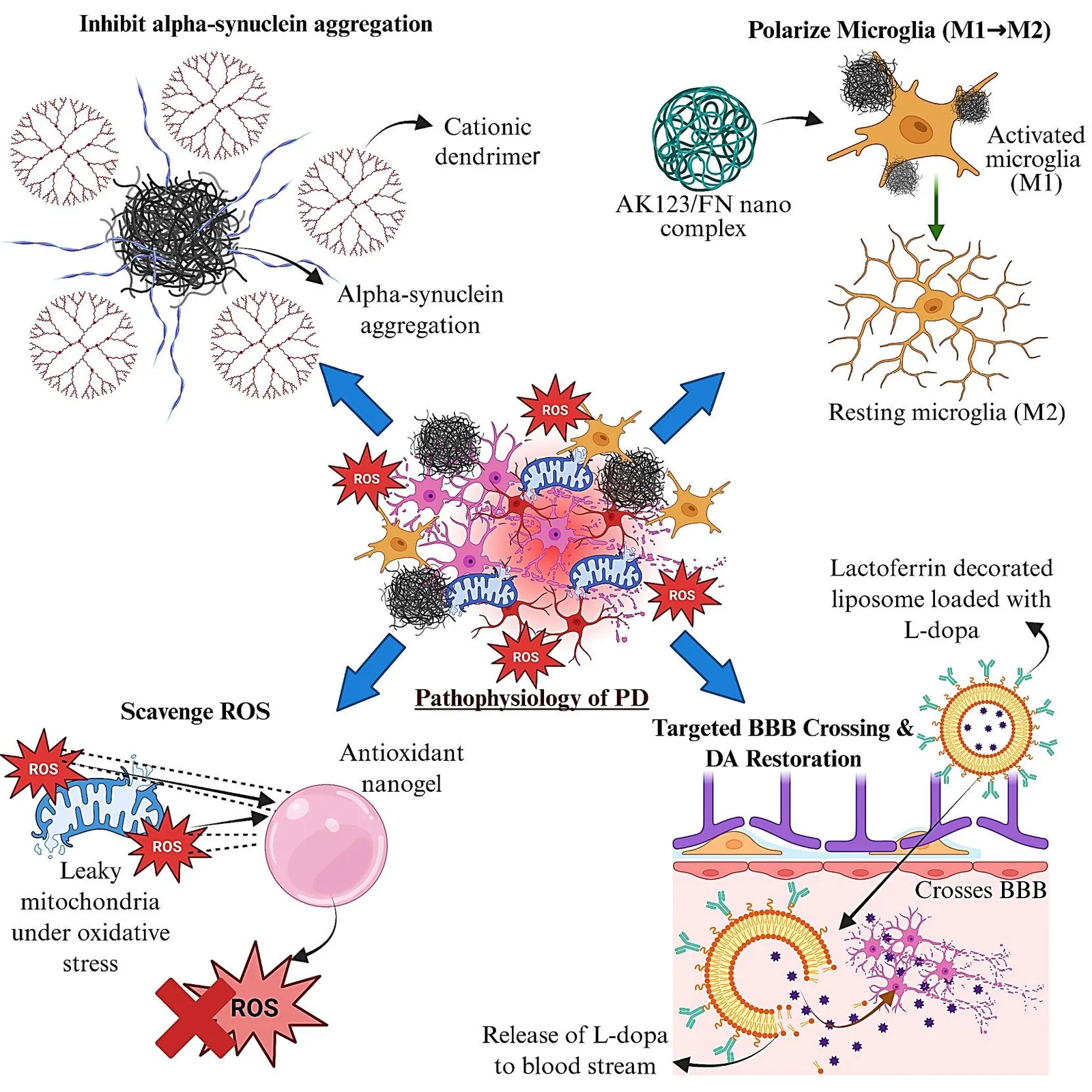
Critical analysis of nanoparticle platforms for PD

###  Lipid-Based Nanoparticles

 Lipid-based nanocarriers such as liposomes and nanolipid carriers (NLCs) also rank among the most popular methods used in nanotechnology. Lipid-based nanocarriers are favoured for their biocompatibility as well as their ability to deliver both hydrophilic as well as lipophilic drugs.^56^

 The versatility of liposomes, a form of nanocarrier studied extensively for PD treatment, has shown promise in intranasal (IN) delivery targeting the brain.^57^ The lipid bilayer composition, a cell-mimicking feature, makes them ideal for drug delivery of various bioactives, ranging from small molecules to more delicate entities such as siRNA. Yet, effective brain delivery is only possible after surface or ligand engineering. For instance, Katamesh et al. (2025) developed albumin-coated liposomes co-delivery systems composed of selegiline and siRNA targeting α-synuclein.^58^ Their IN delivery was more stable with a three-fold enhanced AUC compared with an intravenous (IV) route in a PD rat model induced with rotenone.49 Not only was its performance beneficial for motor function, but restored dopamine levels and inhibited α-synuclein aggregation, thus establishing the advantage of continuous use for targeted therapy via nose-to-brain transport. In contrast, Guo et al. (2025) used a different targeting platform employing a separate liposome for co-delivery of levodopa and curcumin.^59^ This strategy, exploited in an MPTP mouse PD model, resulted in a significant neuroprotective action and a reduction in neuroinflammation, thereby decreasing oxidative stress compared with administration of individual drugs alone. Further, surface-modifying approaches, as exemplified with LMW-Protamine-lactoferrin-coated liposomes, significantly improved neurobehavioral scores and further enhanced their uptake in the brain in preclinical studies, as evidenced in the study by Cong et al. (2025).^60^ The study points toward a profound advantage with surface-modified liposomes advancing their role in non-invasive multidrug therapy targeting. Indeed, their generation emphasises a strict tandem targeting between drug cargos and their targeting designs specialising uniquely in mitigating a specific target, either advancing their PK targeting profiles and/or their multipronged neuroprotective actions.

 Solid lipid nanocarriers (SLNs) are a reliable way to combat poor bioavailability in the brain and the challenge of dosage frequency in the treatment and management of PD, by enabling IN delivery to the brain. The main property of the solid lipid cores in nanocarriers is their high encapsulation capacity and sustained release, which are effective for controlling chronic symptoms of PD. The use of nanocarriers for Parkinson’s treatment is exemplified in the work of Castellani et al. (2024), whereby the nanocarriers consisting of an equal dose of citicoline and dopamine exhibited good *in vitro* drug release and compatibility with neuronal cells.^61^ Comparable results for IN delivery in Parkinson’s treatment are found in the work of Uppuluri et al. (2021), whereby an equal amount of drug (piribedil) was delivered through a thermoresponsive gel in intranasally applied nanocarriers in a rat model, which had an improved four-fold higher in brain drug concentration compared to its systemic exposure, summarised in Table 2.^62^ Efficacious results are also evident in the work of Almeida et al. (2025), whereby S-Carboxymethyl-L-cysteine-loaded SLNs significantly improved motor function and reduced oxidative stress in rotenone-induced PD models and in zebrafish models.^63^

**Table 2:** Summary of comparative analysis of key preclinical studies on lipid and polymeric nanoparticles for PD

**Sl no.**	**Nanoformulation**	**Therapeutic Agent(s)**	**Experimental model**	**Route of administration**	**Key outcomes**	**Ref**
**Lipid nanoparticles **						
1.	Albumin-coated liposomes	Selegiline and α-synuclein siRNA	Rotenone-induced PD rat model	IN route	3-fold increase in brain AUC compared to IV delivery. Improved motor function, restored dopamine and reduced α-synuclein aggregation.	^58^
2.	Liposomes (Co-delivery system)	Levodopa and curcumin	MPTIP-induced PD mouse model	IN route	Significant neuroprotection. Reduced oxidative stress and neuroinflammation compared to monotherapy.	^59^
3.	LMW Protamine / Lactoferrin modified liposomes	Bromocriptine	Haloperidol-induced PD mouse model	IN route	Increased brain targeting efficiency. Enhanced behavioural recovery and neuroprotection.	^60^
4.	SLNs	Dopamine and citicoline	*In vitro* (SH-SY5Y cells)	-	Sustained drug release profile. High cytocompatibility with neuronal cells.	^61^
5.	SLN-loaded thermogel	Piribedil	PD rat model	IN	4-fold increase in brain AUC. 27% direct transport % (DTP). Reduced systemic exposure.	^62^
6.	SLN	S-carboxymethyl-L-cysteine	Rotenone-induced rat PD model and zebrafish model	Likely oral/IN	Improved locomotor activity. Reduced oxidative stress biomarkers and Lewy body formation.	^63^
7.	NLCs	Dopamine	Rotenone-induced PD rat model	IN	~95% entrapment efficiency. *Ex vivo* permeability is 2x higher than the pure drug. Improved motor activity and neurochemistry.	^64^
8.	Chitosan-coated NLCs	Tanshinone IIA	Rotenone-induced PD rat model	IN route	Superior antiparkinsonian and antidepressant effects compared to uncoated NLCs and free drug.	^65^
9.	NLCs	Roflumilast	Preclinical PD model	Oral	Sustained release over 24 hours. Significantly reduced behavioural deficits and neuroinflammation compared to pure drug	^67^
**Polymeric Nanoparticles**						
10.	PEG-PTMC NPs (GB-PPNPs)	Ginkgolide B (GB)	PD mouse model (MPTP/MPP +)	Oral	Sustained release over 48h *in vitro.* Higher drug concentration in plasma and brain compared to free GB. Improved motor activity, restored dopamine.	^32^
11.	PEG-PCL NPs	GB	Zebrafish and rat PD models	Oral	Crossed the BBB in zebrafish. Superior brain concentration and therapeutic efficacy compared to free GB in the rat model.	^69^
12.	Neuromelanin-inspired coordination polymer (DA-NCPs)	Dopamine	*In vitro* BE(2)-M17 cells *In vivo *Rat PD model	IN (Equivalent to 200 µg free DA/day)	~60% drug loading efficiency. Enhanced cellular uptake *in vitro*. Significant improvement in motor symptoms (↓ apomorphine rotations) vs. vehicle & free DA.	^70^
13.	GSH-modified PEO-PCL Diblock copolymer NPs	Levodopa and curcumin	*In vitro *Vero and PC12 cells	-	Stable formulation. Biocompatible with neuronal cells. Designed for GSH-receptor-mediated BBB targeting.	^71^

 NLCs, a next-generation form of LNP, combine a liquid lipid with a solid core to increase drug payload capacity and stabilise it even better than SLNs. NLCs have been developed to accommodate a wide range of therapeutic agents. Additionally, there have been various drug administration routes developed. For IN delivery, Neha et al. (2023) developed dopamine-loaded NLCs. The NCLs demonstrated ~95% entrapment efficiency, along with interestingly high *ex vivo* permeability, which was twice that of the free drug.^64^ Rather impressively, improvements in motor function and biochemical parameters in a rotenone-induced rat model of Parkinson’s disease were observed. Another advantage of NLC-based carriers is that they can incorporate multiple drugs or multi-therapeutic agents. For instance, Hassan et al. (2024) developed NLCs coated using chitosan encapsulated with Tanshinone IIA, which showed higher therapeutic efficiency than uncoated NLCs as well as free drug.^65,66^ NLCs have been effectively used to enhance the oral bioavailability of lipophilic drugs. Another instance of NLC-based delivery systems being effectively used for neurological diseases. Roy et al. (2025) revealed that NLCs encapsulated with roflumilast significantly promoted the sustained release of the drug over a period of 24 hours. Orally administered, NLCs notably reduced behavioural deficits, along with regular neuroinflammatory changes in preclinical models compared to pure drug.^67^ Overall, NLC-based delivery systems have been effectively used to counter poor solubility along with reduced availability to embolic regions in PD.

 Collectively, the evidence supports the lipid NPs as a versatile platform. However, direct comparisons between different lipid carriers that transport identical drugs with identical doses are scarce, making it difficult to rank them definitely in order of preference that arises from more direct comparisons than mere mentions. Such a preference is necessarily contingent on balancing drug properties with delivery route-related expediencies.

###  Polymeric Nanoparticles

 Polymeric nanoparticles are one of the most adaptable nanocarriers that can be used for the treatment of PD. These nanocarriers are made of biodegradable and biocompatible polymers, have an unprecedented ability to control their physical and chemical properties as well as the drug release profiles. The ability to easily produce polymeric nanocarriers for the sustained drug delivery to the brain can be considered a significant remedy for the principal shortcomings of PD therapies, such as low bioavailability and high elimination rates. The most significant benefit of polymeric nanocarriers is their capacity for sustained drug release, which is essential for maintaining drug levels within the brain.^68^

 For example, the research of Zhao et al. (2020) demonstrated that the sustained release of Ginkgolide B (GB) from poly (ethylene glycol)-b-poly (trimethylene carbonate) NPs (GB PPNPs) was achieved over a period of 48 hours.^32^ Indeed, the sustained release of GB led to increased concentrations of GB and its transport across the BBB in the plasma and brain following oral administration in a PD mouse model. Similar observations were obtained when polyethylene glycol-poly(ɷ-caprolactone) NPs were prepared by Wang et al. (2022) loaded with GB with a similar structure to the PEG-PCL nanoparticles, which demonstrated the superior ability to cross the BBB along with improved therapeutic concentrations in a zebrafish and rat model following oral drug administration.^69^ These findings highlight that sustained release from biodegradable polyesters is a reproducible strategy for improving oral bioavailability and neuroprotective efficacy of challenging compounds like GB.

 Polymeric nanoparticles offer far more than simple encapsulation; they provide stabilisation and drug delivery for sensitive drugs. A relatively innovative approach reported by García-Pardo et al. (2021) leveraged neuromelanin-inspired polymeric NPs to create a polymer matrix that could encapsulate dopamine with a loading efficiency of approximately 60%.^70^ These dopamine-loaded NPs not only protected dopamine from degradation and improved dopaminergic cell uptake *in vitro*, but also demonstrated superior efficacy in animal models. In rat models of PD, IN administration exhibited significant enhancement of motor symptoms compared to vehicle and free dopamine administrations, validating a direct dopamine replacement strategy that bypasses the metabolic limitations of levodopa. This indicates both target-specific delivery relative to dopamine and validation for a dopamine replacement strategy that bypasses levodopa’s metabolic limitations.

 Active targeting brings an extra level of complexity with surface modification. As an example, Mogharbel et al. (2022) co-encapsulated levodopa and curcumin with glutathione (GSH) functionalized copolymer NPs in 2022.^71^ They utilise the GSH transporter system at the BBB for active targeting, with promising results indicating that their formulation is stable with low neurotoxicity, proving targeted delivery by the combination approach. Although biocompatibility at the *in vitro* level appears promising, the targeting efficiency at the *in vivo* level with a comparable benefit over non-targeted counterparts has yet to be strongly measurable for most actively targeted systems.

 In conclusion, polymeric NPs provide a versatile delivery system for Parkinson’s disease. As shown in Table 2, there have been success reports for oral and IN delivery, though the degree of benefits depends on drug and polymer combinations and models used.^72^ Future advancements in the field are likely to lead to intelligent polymers and ligands, which are expected to further expand the possibilities for these nanocarriers.

###  The Critical Role of the Administration Route

 The success of any NP therapeutic relies to a great extent on the manner of its delivery. Every route of drug delivery has its own advantages as well as limitations, which also affect the NPs.^73^ Among all routes, the IV route is the most popular because, within a short period, the drug substances can easily be distributed within the body’s circulatory system.^74^ However, the advantage of extensive exposure to body cells raises the likelihood of targeting unintended sites and facilitates rapid elimination by mononuclear phagocytic cells before the drug can reach the target area, i.e., the brain. Methods such as PEGylation can help to delay elimination; however, the ability of the BBB to accept the drug by the IV route is still a basic challenge.^75^

 Due to such limitations, IN delivery routes have been considered for direct CNS delivery. This route exploits the anatomical link between the nose and CNS *via* the olfactory and trigeminal nerves, thereby bypassing the BBB.^76^ The nose-to-brain pathway presents several significant advantages, including a substantial reduction in systemic exposure, which usually of peripheral toxicity and facilitates preferential delivery to the CNS *via* the olfactory bulb, which is implicated in early Parkinsonian lesions.^77^ Mucosal clearance by the nasal epithelium represents one of the main obstacles to this route.^78^ This issue could potentially be resolved or mitigated through the application of suitable mucoadhesive coatings, including chitosan polymer-based formulations or *in situ* gelation systems. Importantly, the clinical translation of IN delivery faces challenges, including high inter-patient variability in nasal anatomy and physiology, mucociliary clearance rates, and the practical limitation of administering high drug doses within a small nasal volume.

 Oral delivery is the most comfortable way for patients to take their medications, but it also has some challenges, like first-pass metabolism, an acidic environment, and the intestinal barrier.^79^ The main benefit of giving NPs orally is that they may protect the drug from breakdown and help it pass through the intestinal barrier into the bloodstream. But to reach the brain, the drug has to get past a few obstacles, like getting through the intestinal barrier, getting into the bloodstream, and finally getting past the BBB. This makes oral delivery less effective than IN delivery for targeting the brain.^80^

 In a nutshell, the route of administration is considered a fundamental step in designing nanoparticles for brain delivery. IN administration is presently one of the most explored options in the case of targeted treatments of PD due to its ability to bypass the BBB. IV and oral routes are still valid choices, although clear limitations and specific therapeutic aims have been considered.

###  Advanced and Specialised Nanocarriers: Multifunctional Platforms

 Beyond simple lipid-based carriers and conventional polymer systems, a new generation of sophisticated nanocarriers has emerged. These platforms introduce novel physicochemical properties that allow for functions such as externally guided targeting and intrinsic therapeutic activity. At the forefront of nanotechnology for PD, these advancements frequently pose increased challenges related to biocompatibility and scalable production.

####  Stimuli-Responsive and Inorganic Nanoparticles 

 Probably the most significant advance in nanomedicine so far is the rise of “smart” nanocarriers, which are rooted in the capacity of such systems to respond to external cues.^81^ Since stimuli-responsive designs and inorganic nanoparticles can be engineered to release their payload or alter behavior in response to certain internal triggers, from acidic pH, high ROS, to overexpressed enzymes, or even to external cues like magnetic fields or ultrasound, they could hence maximise effectiveness at the target site while reducing off-target effects and thereby provide a powerful toolkit against complex diseases like PD.

 Superparamagnetic iron oxide nanoparticles (SPIONs) have many active and synergistic functions as drug carriers. You can not only enable magnetically-guided cell therapy, but you can also track them non-invasively. These two traits make them a good choice for treating PD. Stem cell therapy is a promising application.^56^ A significant challenge in the treatment of PD is ensuring that grafted cells specifically target the regions that have been predominantly impacted. Kim et al. (2021) demonstrated that human adipose-derived stem cells (hASCs) marked with magnetic nanoparticles could be successfully tracked to target areas in a mouse model of PD induced by 6-OHDA. Magnetically targeted cell delivery (MTCD) could make the targeted transfer of these cells even better. These two methods have been separately and successfully tested on PD models. A significant issue is limiting the potential neurotoxic effects on cells once they reach targets. This is particularly relevant if its application is optimised through MTCD. In a rat PD model, Simorgh et al. (2021) applied their alginate-coated SPIONs via IN route and guided them magnetically to the damaged striatum. In their study, this resulted in enhanced retention within the target area, improved motor functions, and a restored dopaminergic system.^82^ Aside from their regenerative uses, magnetic NPs can also aid in advancements in diagnostics. The magnetic core is used as an ultrasensitive biosensor, such as in s-MARSA, as illustrated by Upadhyay et al. (2023), which enables the detection of biomarkers even in low CSF volumes.^83^ It may find its utilisation in monitoring pharmacotherapy and its management of adverse reactions, emphasising its importance in PD management as a diagnostic tool. However, for clinical translation, some issues need to be addressed regarding possible MRI artefacts, the metabolism of iron, its retention levels within the body, as well as the achievable depth of external magnetic targeting within the bodies of human patients.

 Gold nanoparticles (AuNPs) have become a principal component of PD diagnostic tools owing to their biocompatibility, versatile surface chemistry, and specific electrochemical properties. AuNPs possess high sensitivity and specificity for a range of PD biomarkers due to these characteristics. They can be utilised by loading them into electrochemical sensors to detect pathological levels of α-synuclein. For instance, the work by Jeong et al. (2025) entailed the development of a sensor that utilised laser-induced graphene gold plating to detect phosphorylated α-synuclein from blood samples and differentiated PD from control samples by analysing levels of the same.^84^ Another such method by Carneiro et al. (2023) utilised AuNPs by electrodepositing gold nanomaterials onto a carbon nanotube surface to form a nanostructured sensor that had a high sensitivity surface area of 0.101 cm^2^.^85^ The sensor had high recovery for α-synuclein even at a lower limit of 4.1 ng/mL and had high recovery rates for α-synuclein from serum.^86^ Apart from PD biomarkers, AuNPs form the basis of other novel diagnostic tools that highlight their significance as high-performance transducers that can improve the stability of biosensors to enable precise measurement of PD biomarkers. In clinical settings for PD diagnostic purposes, such biosensors need to be completely analytically validated to determine LoDs and LoQs while accounting for complex biological samples such as blood and CSF to gain regulatory approval as a medically valid diagnostic aid.

 Mesoporous silica nanoparticles (MSNs) have been found particularly appealing due to their ability to combine a solid structure with a large surface area and tunable pore topologies. These unique properties also make them highly desirable for targeted drug delivery systems in PD. The NP’s “honeycomb” structure is studded with dense pores that allow it to have a large payload and facilitate its controlled release. A major improvement reported recently by Morales et al. (2021) involves using the drug itself as the controlling component, thereby avoiding drug loading after synthesis and removing the subsequent surfactant material.^87^ In their study on L-DOPA and MSNs, they reported that it is possible to have a controlled drug release triggered by changes in pH. While this is low in the acidic gastric environment to preserve the drug from early release and subsequent loss in the body, it is maintained as a constant and steady process as it enters the intestinal system and is reflected in constant plasma concentrations that facilitate management of motor fluctuations associated with PD. Apart from this innovative synthesis method, the surface chemistry of silica NPs can be designed for improved delivery and efficacy. García et al. (2023), for instance, designed carboxymethylated silica NPs for the delivery of silybin, a neuroprotectant with low water solubility.^88^ In an MPTP-induced mouse PD model, the oral administration of silybin-loaded NPs showed enhanced neuroprotection, with approximately 72% striatal dopamine levels maintained, outperforming oil formulations and a different polymer formulation. Such examples illustrate how the modification of the surface chemistry of silica NPs can increase the bioavailability and therapeutic outcome of challenging drugs. In general, the ability of silica NPs to offer high loading capacity, controlled release properties, and versatile surface properties makes them a versatile and powerful system. They can be considered for symptomatic therapy approaches using drugs such as levodopa, and for the role of silica NPs to boost the delivery of neuroprotection drugs. One critical translational point to remember is that the long-term brain circulation and biodegradation properties of MSNs remain an important area of research for long-term neurological treatments.

####  Macromolecular and Hydrogel-Based Carriers

 In addition to inorganic nanosystems, soft matter nanocarriers are available with a plethora of advantages owing to their sponge-like, strongly interconnected network. Macromolecular and hydrogel-inspired nanocarriers, such as dendrimers and nanogels, can accommodate a vast quantity of drugs and are more biocompatible because they provide an aqueous microenvironment for sensitive drugs by mimicking the biological membranes, which is a key advantage compared to lipid nanocarriers.

 The advent of dendrimers is a paradigm shift in the manner in which we are accustomed to designing nanocarriers. The fact that they are more than a mere drug delivery system is that they themselves hold the potential to treat diseases. The highly branched, symmetrical structure, coupled with the compact surface groups, makes it capable of engaging targets on a molecular level. Talking about their brilliant use is their direct implication in the pathophysiology of PD. Ferrer-Lorente et al. (2022) proposed cationic carbosilane so-called “bow-tie” dendrimers that efficiently suppressed α-synuclein aggregation in a very pertinent human model.^89^ Additionally, individualised multimodal treatment is achievable with dendrimers. In this connection, a study published by Dai et al. (2024) demonstrated that phosphorus dendrimers (AK123) covalently conjugated with fibronectin successfully formed nanocomplexes that could scavenge ROS, induce anti-inflammatory polarisation, and suppress the NF-κB signalling pathway.^90^ Importantly, these nanocomplexes could successfully pass through the BBB, decrease α-synuclein aggregates and restore dopamine levels in Parkinsonian rats. These observations place successful applications of dendrimers within the realms of modifiable nanoplatforms that target specific areas affected by disease.

 Nanogels are small, cross-linked three-dimensional polymers that expand in the presence of water, forming a protective sponge-like framework for drug delivery. They are capable of entrapping and protecting the drug cargo, thereby imparting improved stability and slow, sustained drug release. Primarily, their use is to combat neuroinflammation. For example, Cao et al. (2025) developed a chitosan nanogel encapsulating resveratrol and selenium and proposed its potential use as an active anti-neuroinflammatory agent, which could decrease the level of oxidative stress and modulate the inflammatory pathway.^91^ Notably, their nanogel formulation altered its protein corona in the blood, thereby enhancing its permeability at the BBB. Nanogels exhibit high biocompatibility with IN delivery, thereby providing an attractive nose-to-brain delivery.^92^ A thermosensitive nanogel system encapsulating albiflorin, proposed by Chen et al. (2022) for IN delivery, selectively accumulated in particular areas of the brain and significantly inhibited inflammation in a rat model of PD.^93^ Beyond synthetic drugs, nanogels are also effective in carrying natural extracts. A nanogel encapsulating seeds of *Macuna pruriens, *a natural source of L-DOPA, was proposed and developed by Chittasupho et al. (2022). The nanogel exhibited encouraging neuroprotection and *in vitro* stability, making it a novel oral treatment option for elderly patients.^94^

 Nanoemulsions are a highly efficient nanocarrier system for lipids, as they improve the delivery of challenging drugs. Its key benefit is the increased solubility, stability, and bioavailability of drugs. Intranasally administered nanoemulsions, in particular, are well suited for the lipids in the nasal mucosa, thus facilitating direct nose-to-brain delivery. Usama Ashhar et al. (2022) have formulated an IN nanoemulsion co-loaded with bromocriptine and glutathione, showing its deep penetration throughout the nasal mucosa with higher levels of drug concentration in the brain than suspensions for a haloperidol-induced PD model.^95^ In addition, nano-emulsions may improve oral bioavailability and efficacy. A cannabidiol nano-emulsion, for instance, orally administered by Santos et al. (2025) to a rotenone-induced PD model, elicited neuroprotective, anti-inflammatory, and antioxidant properties through a significant reduction in α-synuclein levels in the brain and recovery in striatal glutathione levels.^96^ Likewise, Geetha et al. (2023) prepared and administered a nano-emulsion formulation of chia seed oil orally, showing significant advancement in neuroprotection compared to the pure drug, with improvements in motor dysfunction and reduced oxidative stress in the rotenone-induced model.^97^

 The diversity of these state-of-the-art nanoplatforms illustrates how nanomedicine is moving from basic drug delivery systems to targeted, sustained, as well as enabling theranostics for PD.

## Challenges and Future Prospects in Nanomedicine Translation

 Although promising outcomes exist in preclinical studies involving animal models, significant translational barriers exist between successful laboratory results and their applicability to patient practice. The key challenges to its clinical application are listed in Table 3.

**Table 3:** Challenges and strategies for translation of nanoparticle therapies in PD

**Sl no. **	**Challenge **	**Impact on translation**	**Potential mitigation strategies**	**Ref. **
1.	Long-term toxicity and biocompatibility	Unknown chronic effects of nanomaterial accumulation in the CNS. Risk of low-grade neuroinflammation and off-target deposition.	Implement microfluidic coaxial flow systems for reproducible nanoprecipitation. Conduct repeat-dose toxicology studies in rodents and non-rodents with specific panels for neuroinflammation (GFAP, IBA1) and detailed histopathology of off-target organs.	^98,99^
2.	Immunogenicity	Risk of CARPA, ABC upon repeated dosing, and hypersensitivity reactions.	Utilise functionalised PEG alternatives like zwitterionic polymers to mitigate anti-PEG immunity. Establish pre-clinical immunotoxicity screening protocols, monitoring IgM/IgG titers and complement activation.	^100^
3.	Bio-corona formation	Non-specific protein absorption in blood plasma can mask targeting ligands, alter cellular uptake, and redirect NPs from their intended brain targets.	Employ “stealth” coating with demonstrated protein resistance (e.g. polysarcosine, dense PEG brushes). Use covalent, site-specific conjugation techniques to ensure ligand orientation and density.	^101^
4.	Scalability and manufacturing	Difficulty in producing large, GMP-compliant batches with consistent particle size, drug loading, encapsulation efficiency and sterility.	Adopt continuous manufacturing platforms (e.g. staggered herringbone micromixers) coupled with real-time PAT for inline monitoring of CQAs.	^102^
5.	Targeting efficiency *in vivo *	Preclinical targeting success in rodent models often fails to translate due to differences in BBB receptor expression, disease pathology and protein corona composition.	Validating targeting in human-relevant models (e.g. iPSC-derived BBB-on-a-chip, cerebral organoids) before animal studies, prioritise ligands with conserved biology, like transferrin and lactoferrin, over species-specific antibodies.	^103^
6.	Regulatory hurdles	Lack of specific, well-defined regulatory pathways for complex nanomedicines creates uncertainty, prolongs development timelines and increases cost.	Engage in early scientific advice meetings with agencies, like the FDA and EMA. Co-develop and adhere to consensus characterisation standards, such as ISO/TS 21387, for size, charge, drug release, and stability.	^104^

###  The Translational Gap: from Animal Models to Human Patients

 The major challenge in PD treatment is the constraints posed by current preclinical research approaches. Present approaches are not very predictive (Figure 3).^105^ Despite showing high therapeutic potential in PD rat models generated using MPTP or 6-OHDA, NPs fail to simulate the expected slow progression observed in PD in humans.^106^ This discordance results in the ‘valley of death’ for drug development, which fails to appropriately simulate outcomes in human trials based on animal studies. Another major aspect is the presence of species-dependent protein variability in blood; the response, uptake, and clearance patterns for NPs in rodents are different from those in humans, thereby reducing the predictability of human results.^107^ Hence, the need for using human-relevant approaches, like microfluidic BBB-on-a-chip systems or brain organoids, is an utmost necessity to bridge these two aspects together for PD.

**Figure 3:**
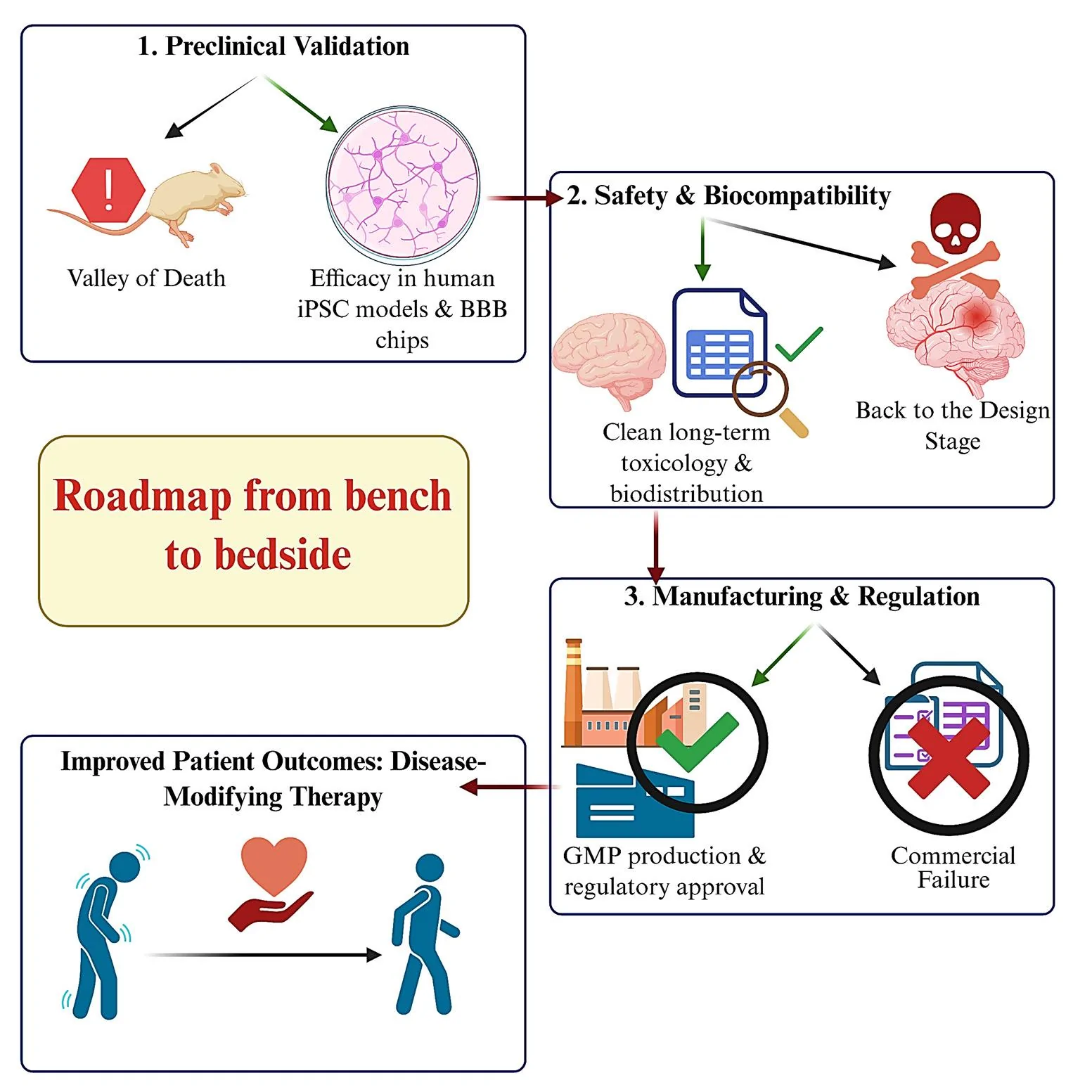
The translational gauntlet: A roadmap from bench to bedside for management of PD

###  Manufacturing and Biocompatibility Hurdles

 The translation of NP therapies from the laboratory to clinical applications depends on solving the significant scaling and biocompatibility problems. Even if NPs are excellent in preclinical studies, manufacturing on a scalable GMP process with good batch-to-batch reproducibility, which is an important requirement, is a persistent challenge.

 The large-scale synthesis of CQAs, such as particle size, PDI, zeta potential, or encapsulation efficiency, involves true technical challenges and high economic costs. Even small changes in these CQAs result in large differences in biodistribution, cellular uptake, and therapeutic efficacy. If a hypothetical PLGA NP formulation for IV administration, for example, is to be accepted in a batch, it should have a particle size distribution of 90-110 nm with a PDI of < 0.1, a zeta potential of -10 to -30 mV, or an encapsulation efficiency > 80% to ensure consistency in PK and toxicity. And there is no doubt that there will be batch-to-batch variability, which poses a serious concern to regulators, not to mention a serious barrier to marketing.

 Despite its applications in manufacturing, biocompatibility is still a concern. Although biodegradable polymers such as PLGA are considered biologically safe, their long-term toxicity profile, especially the activation of microglia leading to low-grade neuroinflammation, the unintentional gradual accumulation within peripheral organs, and unpredictable immunotoxicity due to the eventual breakdown products, remains unclear. Hence, detailed long-term toxicity studies would be required, which would involve the evaluation of systemic toxicity, along with neuroinflammation markers (such as GFAP and IBA-1) within the target areas of the brain, followed by a detailed histopathological assessment of the non-target tissues.

###  The Regulatory and Economic Landscape

 Transitioning from the research laboratory to the clinical bedside has been one of the most challenging steps for product developers.^103^ The way to get regulatory approval is still unclear because agencies like the FDA and EMA are still working on their requirements. Regulators want a lot of data, both *in vitro* and *in vivo*, beyond just purity and potency. This means that properties like particle size and surface chemistry need to stay the same, with a focus on the safety of the nanoformulation.^104^ This is a very loud and time-consuming process that makes development much more expensive.^108^ For the final product for PD to be successful in the market, it must demonstrate a significant therapeutic advantage over existing alternatives like generic levodopa to justify its premium price, secure insurance reimbursement, and ensure patient accessibility. This scary economic reality is a big threat to the success of many promising nanotherapies.

###  Future Perspectives and Strategic Directions

 Future studies need to pursue solutions that are designed with strategy and capable of clearing the path into clinics.

- Construction of “intelligent” carriers: The next generation of nanocarriers should be multi-functional and responsive. Carriers may be designed through surface engineering with targeting ligands such as lactoferrin or transferrin, or even using surface modifiers that respond to other stimuli like elevated ROS, holding much promise for clinical success.^109^
- Combining diagnosis and therapy: Theranostic NPs can integrate an imaging agent (MRI or nuclear imaging) with the therapeutic payload. Thus, clinicians will receive important information on targeted delivery, biodistribution, and drug retention at the site of action, with feedback on the right dose and the right patient, and early evidence that the therapy is working.^110^
- Promoting pre-competitive collaboration: Cross-disciplinary collaboration will hasten the process of translation. Academia, industry, and regulators should collaborate early in product development, from harmonising protocols to shaping regulatory pathways tailored to the unique challenges of nanomedicine.^111^

In all, translation of nanomedicine from the laboratory to real-life care in PD is challenging, yet possible. To fully realise its potential, what must be done is to follow a focused, human-centred approach: use models that genuinely reflect people, ensure strong and scalable manufacturing, and plan regulatory steps early and openly. In that way, we can open more doors to breakthrough treatments for PD.

## Biocompatibility and Toxicity: A Pivotal Hurdle to Clinical Translation

 Although NPs hold much promise, their long-term biocompatibility and potential toxicity remain significant concerns, counteracting the positives. We have to consider these risks as a centrepiece for evaluation, and not an afterthought in translating from bench to bedside. The toxicity profile of an NP should be taken into consideration about the physicochemical characteristics of the carrier, namely size, shape, and surface charge, since these factors control the site where particles are distributed within the body, their stability, and most importantly, their long-term safety during PD management.

###  Mechanisms and Manifestations of Neurotoxicity

 Oxidative stress and long-term brain inflammation are still major safety concerns for particles that act inside the brain. Some NPs are intended to prevent oxidation, but others, especially some metal-based ones, can accidentally produce ROS through reactions on their surfaces. Exacerbation of neuronal damage and trigger can occur in a brain that is already under oxidative stress due to PD pathology, leading to disease worsening. This is a critical double-edged sword. While some studies show promising results to suppress neuroinflammation using NPs such as dendrimers, a poorly designed NP can cause the opposite action, potentially fuelling the disease progression.^112^

 The ability of NPs to cross the BBB can be both good and bad. We still don’t know how long-term accumulation in brain tissue works. There is a clear need for long-term toxicology studies to learn more about how biodegradable polymers like PLGA breakdown products might affect healthy neurons and how they move through and out of the brain.^113^

###  Mitigation Strategies and the Route to Safer Design

 These challenges have, in turn, driven the development of sophisticated approaches that consider safety as a central parameter even from the initial design phase.^114^

- Surface engineering for stealth and targeted delivery: Coating NPs with hydrophilic polymers, such as PEG, minimises opsonisation and hence reduces the nonspecific immune recognition process often referred to as a “stealth” effect. However, during long-term therapeutic procedures, this approach needs to be balanced against the potential for accelerated blood clearance mediated by anti-PEG immune responses.^115^ The use of targeting ligands, such as lactoferrin or transferrin, promotes drug uptake at the site of action while limiting off-target exposure and toxicity.
- The important role of rigorous characterisation: The relationship between physicochemical properties and toxicity is not merely theoretical. Clinical translation may be significantly hampered by a variable safety profile of the NPs, depending on their size or surface charge. Thus, extensive *in vitro* screening in relevant cell lines needs to be performed, followed by long-term *in vivo* biodistribution and toxicology studies in adequate PD models, to take nanotechnology further forward in PD.^116^

The key message is not to stop using nanocarriers but, rather, to design these nanocarriers with safety issues in mind. In other words, starting with a “safety-by-design” approach in which biocompatibility is a key driver from early formulation onwards, combined with the comprehensive characterisation and manufacturing controls described above.

## Conclusion

 Nanotechnology is progressively influencing a treatment strategy for PD. With the help of nano-based drug delivery systems for PD treatment, we can easily target key problems related to crossing the BBB and overcoming conventional therapies. This review critically evaluates preclinical studies available in animal models for a range of nanotherapeutics with a focus ranging from simple lipid-based nanoparticles to more advanced “smart” delivery systems such as dendrimers and inorganic NPs. However, to realise this promise from the laboratory to the clinic, some challenges need to be overcome. It is crucial to address issues such as manufacturing on a scalable basis, working on relevant models that can mirror the diseases affecting humans, and knowing the toxicity levels on a long-term basis. Simple nanocarriers are no longer the future prospects of PD nanomedicine. Intelligent, multi-functional systems that can deliver a combined targeted therapy, with real-time diagnostic capabilities, are considered the future in the management of PD. By addressing these translational hurdles through interdisciplinary collaboration between researchers, clinicians, and regulators, we have a genuine opportunity to improve the prognosis of patients with PD using nanotechnology for both symptomatic management as well as disease-modifying therapy.

## Competing Interests

 The authors declare no financial or other conflicts of interest related to this work.

## Ethical Approval

 Not applicable.
